# Supplementary material for: Antiplasmodial Activity of Vachellia xanthophloea (Benth.) P.J.H. Hurter (African Fever Tree) and Its Constituents
Source: Pharmaceuticals (Basel). 2022 Apr 13;15(4):470. doi: 10.3390/ph15040470 (PMC9033093; doi:10.3390/ph15040470)
Supplement: Supplementary file 1 [file pharmaceuticals-15-00470-s001.zip › pharmaceuticals-1619319-supplementary.pdf]

Supplementary materials

Antiplasmodial activity of *Vachellia xanthophloea* (Benth.) P.J.H. Hurter (African fever tree) and its constituents

Nasir Tajuddeen[1], Tarryn Swart[2], Heinrich C. Hoppe[2], and Fanie R. van Heerden[1,*]

[1] School of Chemistry and Physics, University of KwaZulu-Natal, Private Bag X01, Scottsville 3209, Pietermaritzburg, South Africa 1; ntajuddeen@yahoo.com, vanheerdenf@ukzn.ac.za

[2] Department of Biochemistry & Microbiology, Rhodes University, Grahamstown 6140, South Africa; g10s2905@campus.ru.ac.za, h.hoppe@ru.ac.za

***** Correspondence: vanheerdenf@ukzn.ac.za (F.R. van Heerden); Tel.: +27 82 823 8642

Abstract: *Vachellia xanthophloea* is used in Zulu folk medicine as an antimalarial remedy. Moderate antiplasmodial activity was previously reported for the plant's extracts against D10 *Plasmodium falciparum*. This study aimed to identify the phytochemicals responsible for the antiplasmodial activity of the leaf extract. The compounds were isolated by chromatography and their structures were determined using spectroscopic and spectrometric methods. The antiplasmodial activity was evaluated using the parasite lactate dehydrogenase assay and the cytotoxicity was determined using the resazurin assay. The ethyl acetate fraction inhibited *P. falciparum* with IC_{50} = 10.6 μg/mL and showed minimal cytotoxicity (98% cell viability at 33 μg/mL). Chromatographic purification of this fraction afforded sixteen compounds, including two new flavonoids. Also, a 1:1 mixture of phytol and lupeol was isolated from the hexanes fraction. All the compounds are reported from *V. xanthophloea* for the first time. Among the isolated metabolites, methyl gallate displayed the best activity against *P. falciparum* (IC_{50} = 1.2 μg/mL), with 68% viability of HeLa cells at 10 μg/mL. Therefore, methyl gallate is responsible for the antiplasmodial activity of *V. xanthophloea* leaf extract and its presence in the leaf extract might account for the folkloric use of the plant as an antimalarial remedy.

Keywords: *Vachellia xanthophloea*; Fabaceae; flavonoids; methyl gallate; malaria; *Plasmodium*

Spectroscopic data of the known compounds:

2′-Hydroxy-3,7,8,4′,5′-pentamethoxyflavone (**2**): pale-yellow solid, UV (MeOH/ACN): λ_{max} 247, 344 nm; ^{1}H NMR (500 MHz, $CDCl_3$): δ_H 8.15 (1H, s, OH), 7.99 (1H, d, *J*=9.0 Hz, H-5), 7.06 (1H, d, *J*=9.0 Hz, H-6), 7.05 (1H, s, H-6′), 6.63 (1H, s, H-3′), 4.00 (3H, s, OCH_3), 3.99 (3H, s, OCH_3), 3.93 (3H, s, OCH_3), 3.92 (3H, s, OCH_3), 3.89 (3H, s, OCH_3). ^{13}C NMR (125 MHz, $CDCl_3$) δ_C 173.4 (C-4), 156.6 (C-7), 155.2 (C-2), 153.7 (C-4′), 151.3 (C-2′), 150.1 (C-9), 143.5 (C-5′), 138.2 (C-3), 136.8 (C-8), 121.1 (C-5), 118.8 (C-10), 114.0 (C-6′), 108.9 (C-1′), 110.1 (C-6), 102.8 (C-3′), 61.5 (OCH_3-8), 61.9 (OCH_3-3), 56.1 (OCH_3-5'), 56.2 (OCH_3-4'), 56.5 (OCH_3-7). HPLC R_t: 24.101 min; HR-ESI-(+)-MS: *m/z* 389.1248 $[M+H]^+$ (Calculated for $C_{20}H_{21}O_8$, 389.1236).

3-*O*-Methylquercetin (**4**): yellow solid, UV (MeOH/ACN): λ_{max} 266, 373 nm; ^{1}H NMR (400 MHz, CD_3OD): δ_H 7.62 (1H, d, *J*=2.1 Hz, H-2′), 7.52 (1H, dd, *J*=8.5, 2.1 Hz, H-6′), 6.9 (1H, d, *J*=8.5 Hz, H-5′), 6.38 (1H, d, *J*=1.9 Hz, H-8), 6.19 (1H, d, *J*=1.9 Hz, H-6), 3.78 (3H, s, OCH_3). ^{13}C NMR (100 MHz, CD_3OD): δ_C 180.0 (C-4), 166.1 (C-7), 163.1 (C-5), 158.4 (C-9), 158.0 (C-2), 150.0 (C-4′), 146.5 (C-3′), 139.5 (C-3), 123.0 (C-6′), 122.3 (C-1′), 116.5 (C-5′), 116.4 (C-2′), 105.8 (C-10), 99.8 (C-6), 94.8 (C-8), 60.5 (OCH_3-3). HPLC R_t: 23.275 min; HR-ESI-(-)-MS: *m/z* 315.0508 $[M-H]^-$ (Calculated for $C_{16}H_{11}O_7$, 315.0505).

Quercetin (**5**): yellow solid, UV (MeOH/ACN): λ_{max} 254, 371 nm; ^{1}H NMR (400 MHz, CD_3OD): δ_H 7.73 (1H, d, *J*=1.9 Hz, H-2′), 7.63 (1H, dd, *J*=8.4, 1.9Hz, H-6′), 6.88 (1H, d, *J*=8.4 Hz, H-5′), 6.38 (1H, d, *J*=1.9

Hz, H-8), 6.18 (1H, d, *J*=1.9 Hz, H-6). ^{13}C NMR (100 MHz, CD_3OD): δ_C 177.3 (C-4), 166.0 (C-7), 162.5 (C-5), 158.3 (C-9), 148.8 (C-4′), 148.0 (C-2), 146.3 (C-3′), 137.2 (C-3), 124.2 (C-1′), 121.7 (C-6′), 116.3 (C-2′), 116.0 (C-5′), 104.5 (C-10), 99.4 (C-6), 94.5 (C-8). HPLC R_t: 22.136 min; HR-ESI-(-)-MS: *m/z* 301.0341 $[M-H]^-$ (Calculated for $C_{15}H_9O_7$, 301.0348).

(*2R,3R*)-Dihydroquercetin ((+)-taxifolin) (**6**): brown solid, $[\alpha]_D^{24.9}$ +34.6 (*c*=0.78, MeOH), UV (MeOH/ACN): λ_{max} 298 nm; ^{1}H NMR (400 MHz, CD_3OD): δ_H 6.96 (1H, d, *J*=1.86 Hz, H-2′), 6.85 (1H, dd, *J*=8.2, 1.86 Hz, H-6′), 6.8 (1H, d, *J*=8.2 Hz, H-5′), 5.92 (d, 1H, *J*=2.2 Hz, H-8), 5.88 (1H, d, *J*=2.2 Hz, H-6), 4.91 (1H, d, *J*=11.5 Hz, H-2), 4.49 (1H, d, *J*=11.5, Hz, H-3). ^{13}C NMR (100 MHz, CD_3OD): δ_C 198.4 (C-4), 168.8 (C-5), 165.3 (C-7), 164.5 (C-9), 147.2 (C-4′), 146.3 (C-3′), 129.9 (C-1′), 120.9 (C-6′), 116.1 (C-2′), 115.9 (C-5′), 101.8 (C-10), 97.4 (C-6), 96.3 (C-8), 85.1 (C-2), 73.7 (C-3). HPLC R_t: 15.856 min; HR-ESI-(-)-MS: *m/z* 303.0517 $[M-H]^-$ (Calculated for $C_{15}H_{11}O_7$, 303.0505).

(+)-Catechin (**7**): brown solid, $[\alpha]_D^{24.9}$ +7.1 (*c*=0.68, MeOH), UV (MeOH/ACN): λ_{max} 279 nm; ^{1}H NMR (400 MHz, CD_3OD): δ_H 6.83 (1H, d, *J*=1.8 Hz, H-2′), 6.76 (1H, d, *J*=8.0, Hz, H-5′), 6.72 (1H, dd, *J*=8.0, 1.8 Hz, H-6′), 5.93 (1H, d, *J*=2.3 Hz, H-6), 5.86 (1H, d, *J*=2.3 Hz, H-8), 4.57 (1H, d, *J*=7.5 Hz, H-2), 3.98 (1H, ,dt, *J*=7.7, 5.3 Hz, H-3), 2.85 (1H, dd, *J*=16.0, 5.5 Hz, H-4a), 2.51 (1H, dd, *J*=16.5, 8.5 Hz, H-4b). ^{13}C NMR (100 MHz, CD_3OD): δ_C 157.8 (C-7), 157.6 (C-5), 156.9 (C-9), 146.2 (C-3′ and C-4′), 132.2 (C-1′), 120.1 (C-6′), 116.1 (C-5′), 115.3 (C-2′), 100.9 (C-10), 96.4 (C-6), 95.5 (C-8), 82.8 (C-2), 68.8 (C-3), 28.5 (C-4). HPLC R_t: 9.039 min; HR-ESI-(-)-MS: *m/z* 289.0706 $[M-H]^-$ (Calculated for $C_{15}H_{13}O_6$, 289.0712).

Gallocatechin (**8**): brown solid, UV (MeOH/ACN): λ_{max} 270 nm; ^{1}H NMR (400 MHz, CD_3OD): δ_H 6.41 (2H, s, H-2′,6′), 5.92 (1H, d, *J*=2.3 Hz, H-6), 5.86 (1H, d, *J*=2.3 Hz, H-8), 4.53 (1H, d, *J*=7.0 Hz, H-2), 3.94 (1H, dt, *J*=7.0, 5.5 Hz, H-3), 2.81 (1H, dd, *J*= 16.0, 5.5 Hz, H-4a), 2.51 (1H, dd, *J*=16.5, 8.5 Hz, H-4b). ^{13}C NMR (100 MHz, CD_3OD): δ_C 157.8 (C-7), 157.6 (C-5), 156.9 (C-9), 146.8 (C-3′ and C-5′), 134.1 (C-4′), 131.7 (C-1′), 107.3 (C-2′, C-6′), 100.8 (C-10), 96.3 (C-6), 95.5 (C-8), 82.8 (C-2), 68.8 (C-3), 28.1 (C-4). HPLC R_t: 6.923 min; HR-ESI-(-)-MS: *m/z* 305.0649 $[M-H]^-$ (Calculated for $C_{15}H_{13}O_7$, 305.0661).

Methyl gallate (**9**): white solid, UV (MeOH/ACN): λ_{max} 271 nm; ^{1}H NMR (400 MHz, CD_3OD): δ_H 7.05 (2H, s, H-2′, 6′), 3.80 (3H, s, H-OCH_3). ^{13}C NMR (100 MHz, CD_3OD): δ_C 169.05 (C=O), 146.44 (C-3 and C-5), 139.73 (C-4), 121.46 (C-1), 110.08 (C-2 and C-6), 52.29 (OCH_3). HPLC R_t: 11.149 min; HR-ESI-(-)-MS: *m/z* 183.0291 $[M-H]^-$ (Calculated for $C_8H_7O_5$, 183.0293).

Kaempferol (**10**): yellow solid, ^{1}H NMR (400 MHz, CD_3OD): δ_H 7.86 (2H, d, *J*=8.8 Hz, H-2′,6′), 6.93 (2H, d, *J*=8.8 Hz, H-3′,5′), 6.38 (1H, d, *J*=2.2 Hz, H-8), 6.17 (1H, d, *J*=2.4 Hz, H-6).

Apigenin (**11**): greenish yellow solid, ^{1}H NMR (400 MHz, CD_3OD): δ_H 7.88 (2H, d, *J*=8.8 Hz, H-2′,6′), 6.96 (2H, d, *J*=8.8 Hz, H-3′,5′), 6.62 (1H, s, H-3), 6.48 (1H, d, *J*=2.1 Hz, H-8), 6.23 (1H, d, *J*=2.1 Hz, H-6).

Pinoresinol (**12**): grey solid, UV (MeOH/ACN): λ_{max} 279 nm; ^{1}H NMR (400 MHz, $CDCl_3$): δ_H 6.89 (1H, d, *J*=1.7 Hz, H-2/2′), 6.88 (1H, d, *J*=8.1 Hz, H-5/5′), 6.82 (1H, dd, *J*=8.1, 1.7 Hz, H-6/6′), 5.6 (1H, s, OH), 4.74 (1H, d, *J*=4.3 Hz, H-7/7′), 4.25 (1H, dd, *J*=9.0, 7.0 Hz, H-9α/9′α), 3.9 (3H, s, OCH_3), 3.88 (1H, *J*=8.5, 3.0 Hz, H-9β/9′β), 3.1 (1H, m, H-8/8′). ^{13}C NMR (100 MHz, $CDCl_3$): δ_C 146.8 (C-3/3′), 145.3 (C-4/4′), 133.0 (C-1/1′), 119.0 (C-6/6′), 114.3 (C-5/5′), 108.7 (C-2/2′), 85.9 (C-7/7′), 71.7 (C-9/9′), 56.0 (OCH_3), 54.2 (C-8/8′). HPLC R_t: 20.707 min; HR-ESI-(-)-MS: *m/z* 357.1332 $[M-H]^-$ (Calculated for $C_{20}H_{21}O_6$, 357.1338).

(*E*)-Lutein (**13**): orange powder, UV (MeOH/ACN): λ_{max} 445, 472 nm; ^{1}H NMR (400 MHz, $CDCl_3$): δ_H 6.67-6.57 (4H, m, H-11, H-11′, H-15, H-15′), 6.35 (1H, d, *J*=14.8 Hz, H-12, H-12′), 6.25 (2H, d, *J*=9.5 Hz, H-14, H-14′), 6.11-6.17 (5H, m, H-7, 8, 8′, 10, 10′), 5.54 (1H, brs, H-4′), 5.43 (1H, dd, *J*=15.2, 9.9 Hz, H-7′), 4.25 (1H, brs, H-3′eq), 4.00 (1H, m, H-3ax), 2.41 (1H, brs, H-6′), 2.39 (1H, brs, H-4eq), 2.05 (1H, brd, H-4ax), 1.97 (9H, s, CH_3-19,20,20′), 1.91 (3H, s, CH_3-19′), 1.84 (1H, dd, *J*=13.2, 5.8 Hz, H-2′eq), 1.78 (1H, brs, H-

2eq), 1.74 (3H, s, CH_3-18), 1.63 (3H, s, CH_3-18′), 1.48 (1H, brs, H-2ax), 1.37 (1H, dd, *J*=13.3, 6.7 Hz, H-2′ax), 1.07 (6H, s, CH_3-16,17), 0.99 (3H, s, H-17′), 0.84 (3H, s, 16′). ^{13}C NMR (100 MHz, $CDCl_3$): δ_C 138.6 (C-8), 137.8 (C-6, 5′), 137.6 (C-12, 12′, 8′), 136.5 (C-13, 13′), 135.9 (C-9), 135.1 (C-9′), 132.6 (C-14, 14′), 131.4 (C-10), 131.0 (C-7), 130.8 (C-10′), 130.1 (C-15, 15′), 128.8 (C-7′), 126.2 (C-5), 125.0 (C-11), 124.8 (C-11′), 124.5 (C-4′), 66.0 (C-3), 65.2 (C-3′), 55.0 (C-6′), 44.7 (C-2′), 42.6 (C-4), 37.1 (C-1), 34.1 (C-1′), 30.3 (C-16), 29.7 (C-2), 29.6 (C-17′), 28.8 (C-17), 24.4 (C-16′), 22.9 (C-20′), 22.7 (C-18′), 21.7 (C-19′), 14.1 (C-18), 13.1 (C-20), 12.8 (C-19). HPLC R_t: 41.795 min; HR-ESI-(+)-MS m/z 568.4295 M^+ (Calculated for $C_{40}H_{56}O_2$, 568.4280).

1-Heptacosanol (**14**): White amorphous solid, ^{1}H NMR (400 MHz, $CDCl_3$): δ_H 3.64 (2H, t, *J*=6.5 Hz, H-1), 1.56 (2H, m, H-2), 1.26 (46H, brs, H-3 – H-26), 0.88 (3H, t, *J*=6.8 Hz, H-27). ^{13}C NMR (100 MHz, $CDCl_3$): δ_C 63.2 (C-1), 32.9 (C-2), 32.0 (C-3), 29.4-29.8 (C4-24), 25.8 (C-25), 22.7 (C-26), 14.2 (C-27).

NMR and MS spectra of the new compounds

Elemental Composition Report **Page 1**

Single Mass Analysis
Tolerance = 5.0 PPM / DBE: min = -1.5, max = 50.0
Element prediction: Off
Number of isotope peaks used for i-FIT = 3

Monoisotopic Mass, Even Electron Ions
2 formula(e) evaluated with 1 results within limits (all results (up to 1000) for each mass)
Elements Used:
C: 20-25 H: 20-25 O: 5-10
NT-V 2_5 12 (0.387) Cm (1:58)
TOF MS ES+

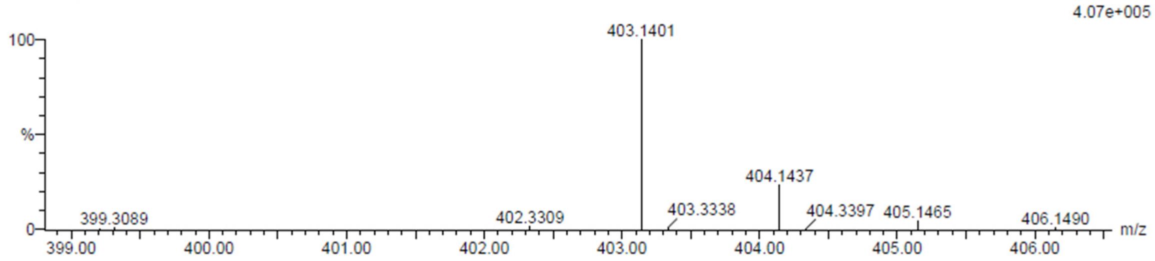

Minimum:				-1.5			
Maximum:		5.0	5.0	50.0			
Mass	Calc. Mass	mDa	PPM	DBE	i-FIT	i-FIT (Norm)	Formula
403.1401	403.1393	0.8	2.0	10.5	47.1	0.0	C21 H23 O8

Figure S1. HRESIMS of 3,7,8,2',4',5'-hexamethoxyflavone (**1**).

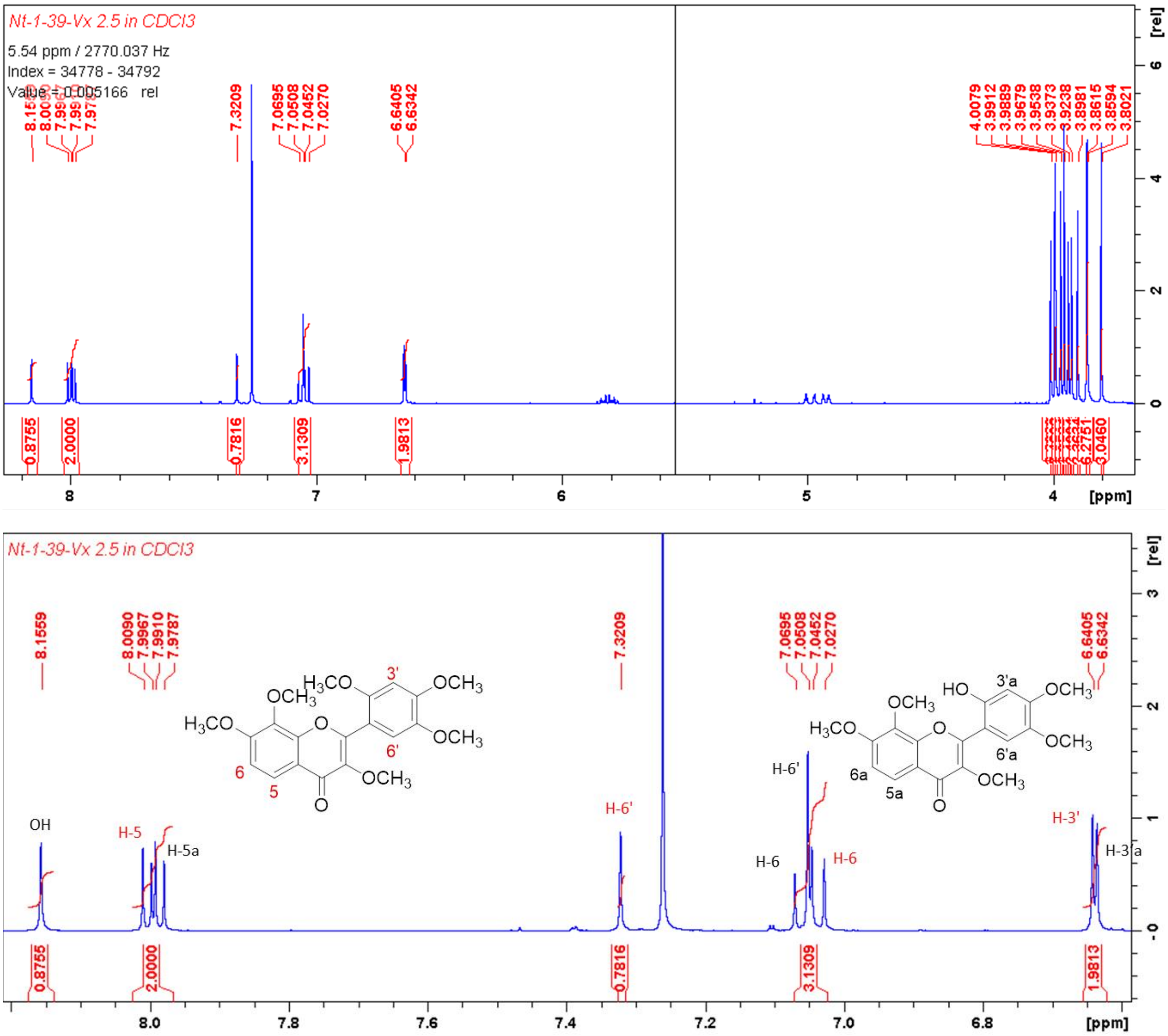

Figure S2. ^{1}H NMR spectrum of 3,7,8,2',4',5'-hexamethoxyflavone and 2'-hydroxy-3,7,8,4',5'-pentamethoxyflavone (**1** and **2**).

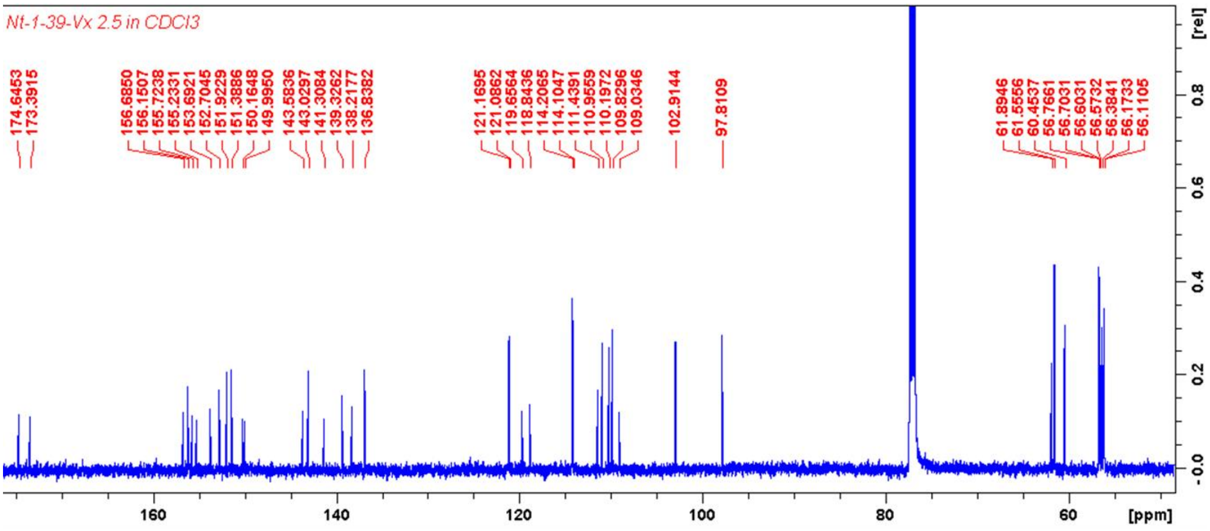

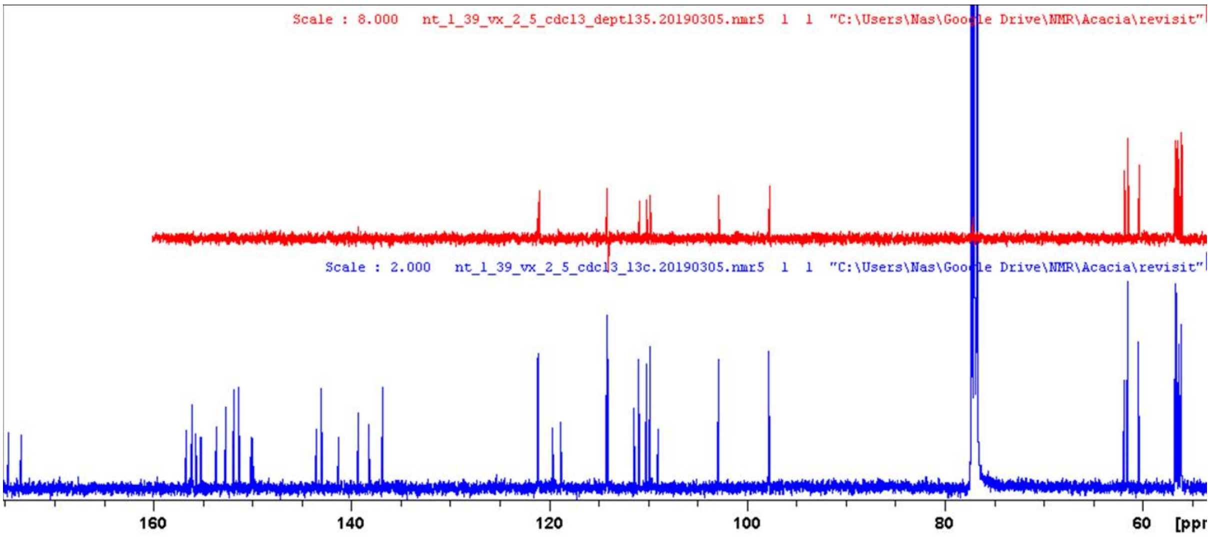

Figure S3. ^{13}C and DEPT spectra of 3,7,8,2',4',5'-hexamethoxyflavone and 2'-hydroxy-3,7,8,4',5'-pentamethoxyflavone (**1** and **2**).

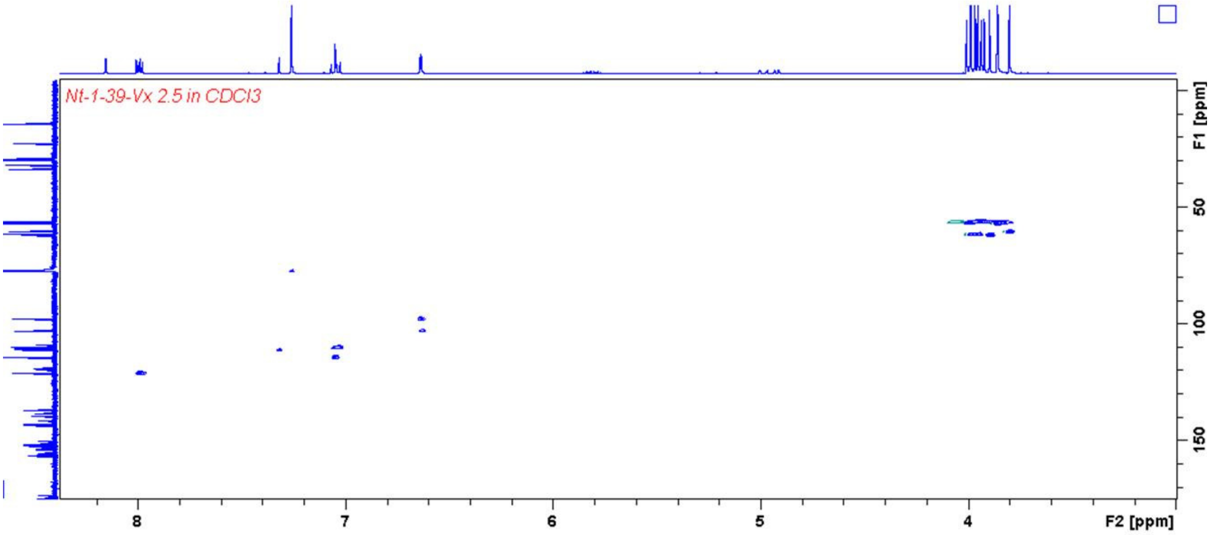

Figure S4. HSQC spectrum of 3,7,8,2',4',5'-hexamethoxyflavone and 2'-hydroxy-3,7,8,4',5'-pentamethoxyflavone (**1** and **2**).

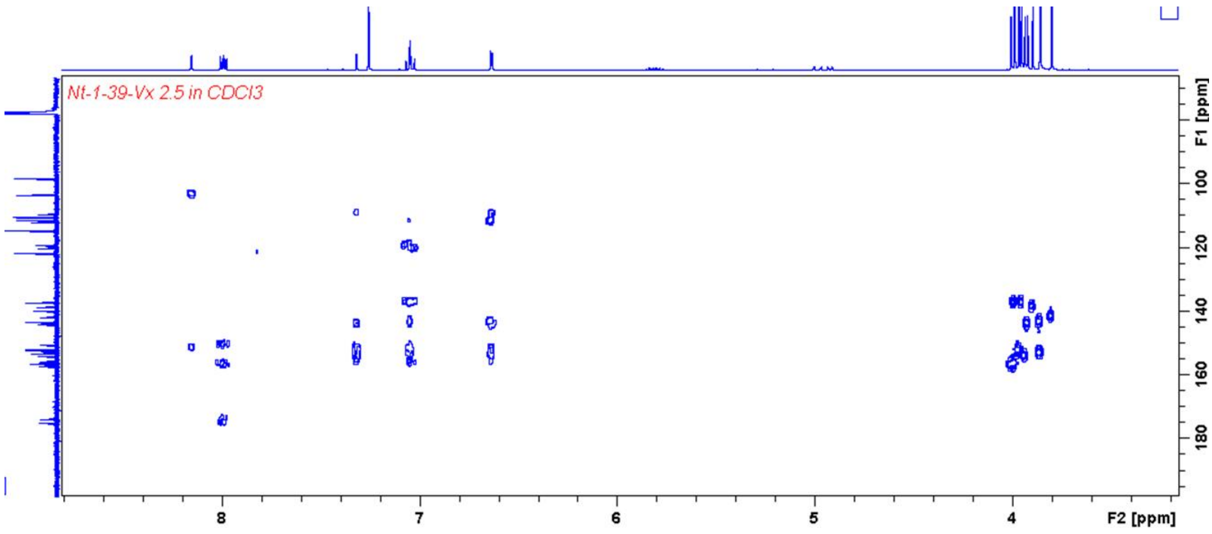

Figure S5. HMBC spectrum of 3,7,8,2',4',5'-hexamethoxyflavone and 2'-hydroxy-3,7,8,4',5'-pentamethoxyflavone (**1** and **2**).

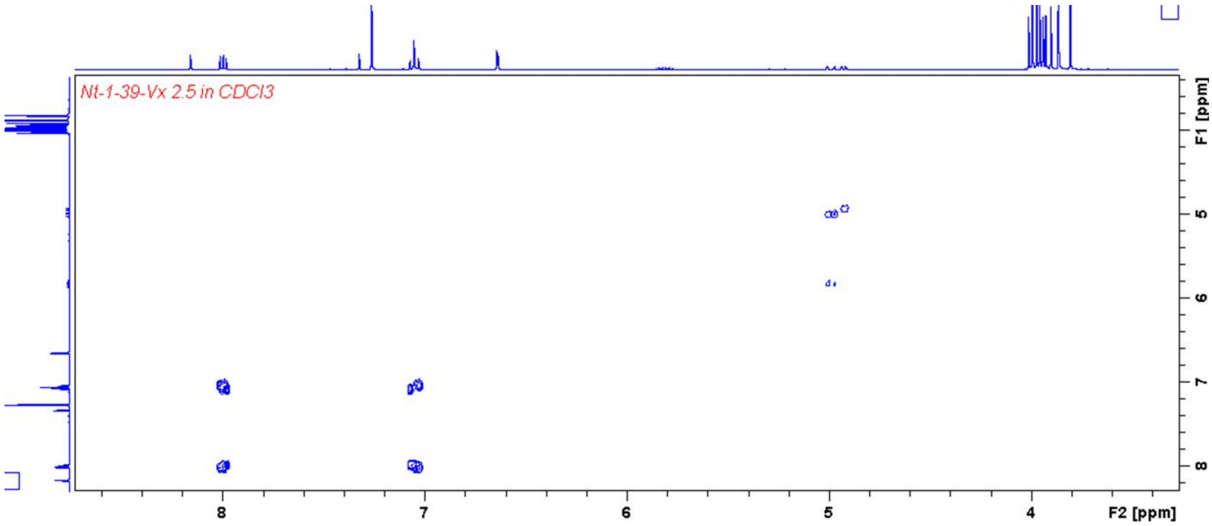

Figure S6. COSY spectrum of 3,7,8,2',4',5'-hexamethoxyflavone and 2'-hydroxy-3,7,8,4',5'-pentamethoxyflavone (**1** and **2**).

Elemental Composition Report

Page 1

Single Mass Analysis

Tolerance = 5.0 PPM / DBE: min = -1.5, max = 100.0
Element prediction: Off
Number of isotope peaks used for i-FIT = 3

Monoisotopic Mass, Even Electron Ions
14 formula(e) evaluated with 1 results within limits (all results (up to 1000) for each mass)
Elements Used:
C: 15-30 H: 15-30 O: 0-10
Vx 2,7b2 32 (1.046) Cm (1:61)
TOF MS ES-

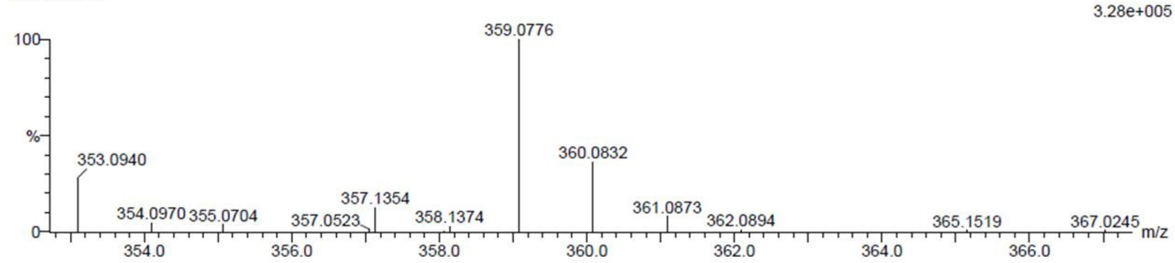

Minimum:				-1.5			
Maximum:		5.0	5.0	100.0			
Mass	Calc. Mass	mDa	PPM	DBE	i-FIT	i-FIT (Norm)	Formula
359.0776	359.0767	0.9	2.5	11.5	33.9	0.0	C18 H15 O8

Figure S7. HRESIMS of 5,7,2'-trihydroxy-3,4',5'-trimethoxyflavone (**3**).

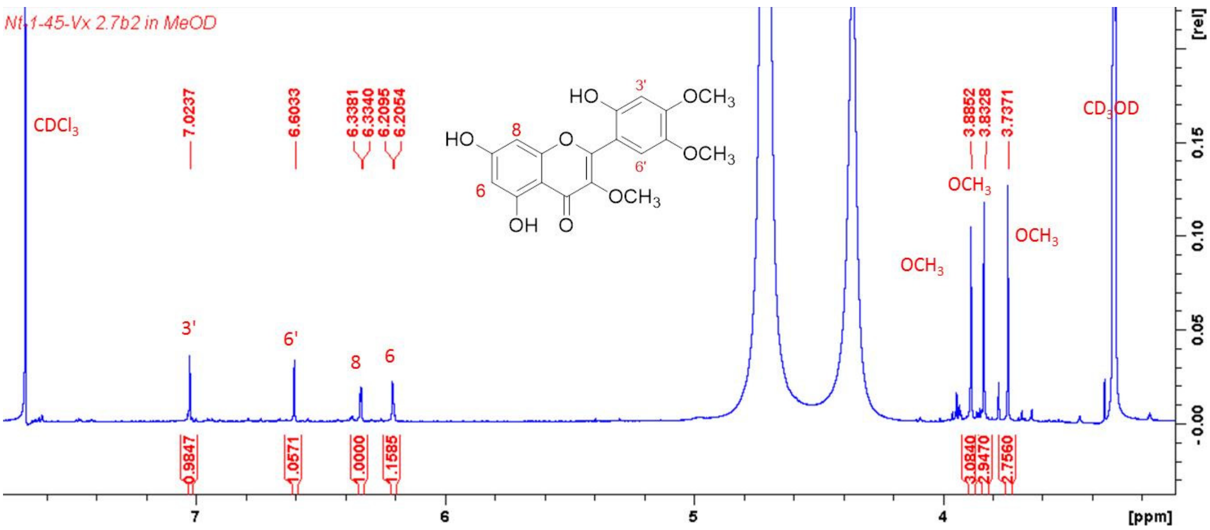

Figure S8. ^{1}H NMR Spectrum of 5,7,2'-trihydroxy-3,4',5'-trimethoxyflavone (**3**).

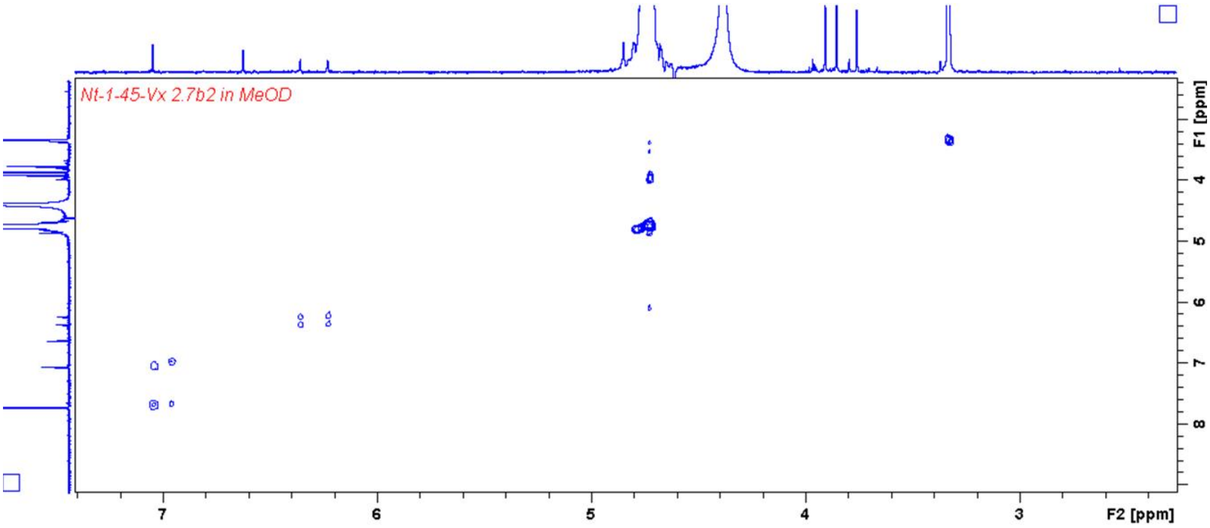

Figure S9. COSY Spectrum of 5,7,2'-trihydroxy-3,4',5'-trimethoxyflavone (**3**).

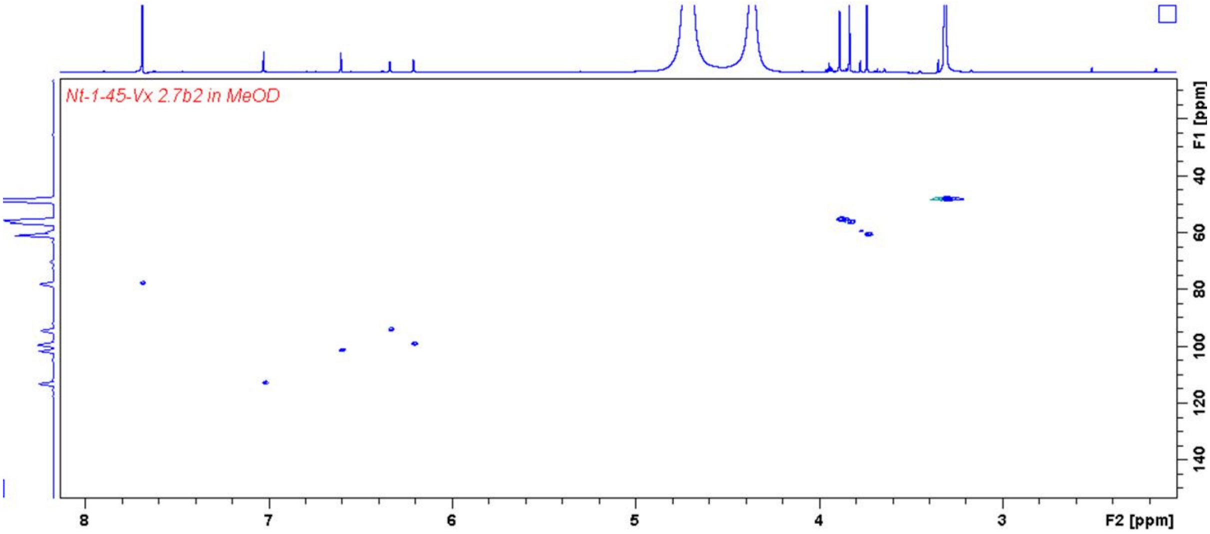

Figure S10. HSQC spectrum of 5,7,2'-trihydroxy-3,4',5'-trimethoxyflavone (**3**).

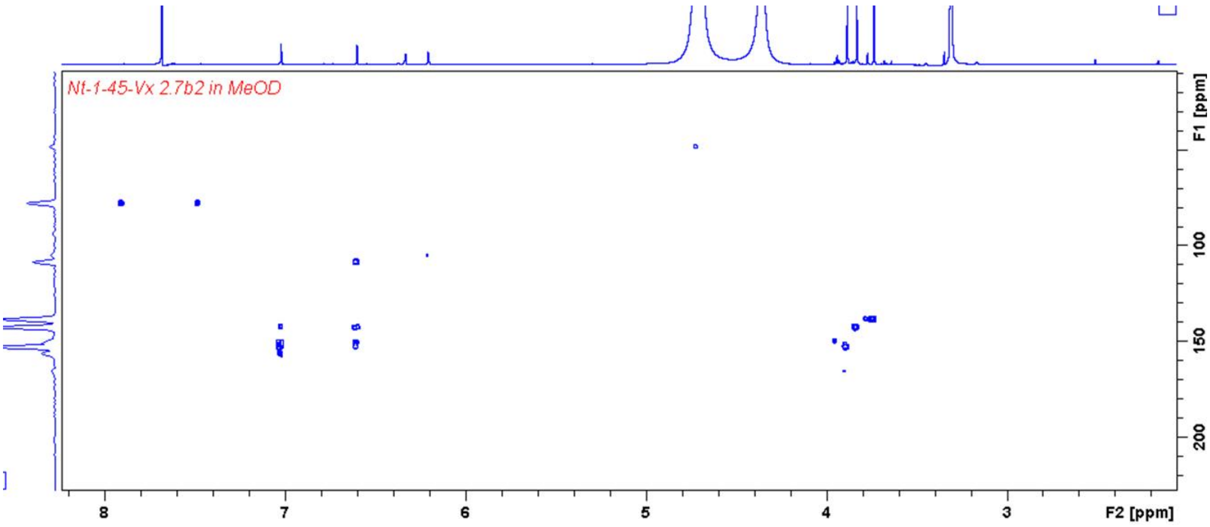

Figure S11. HMBC Spectrum of 5,7,2'-trihydroxy-3,4',5'-trimethoxyflavone (**3**).

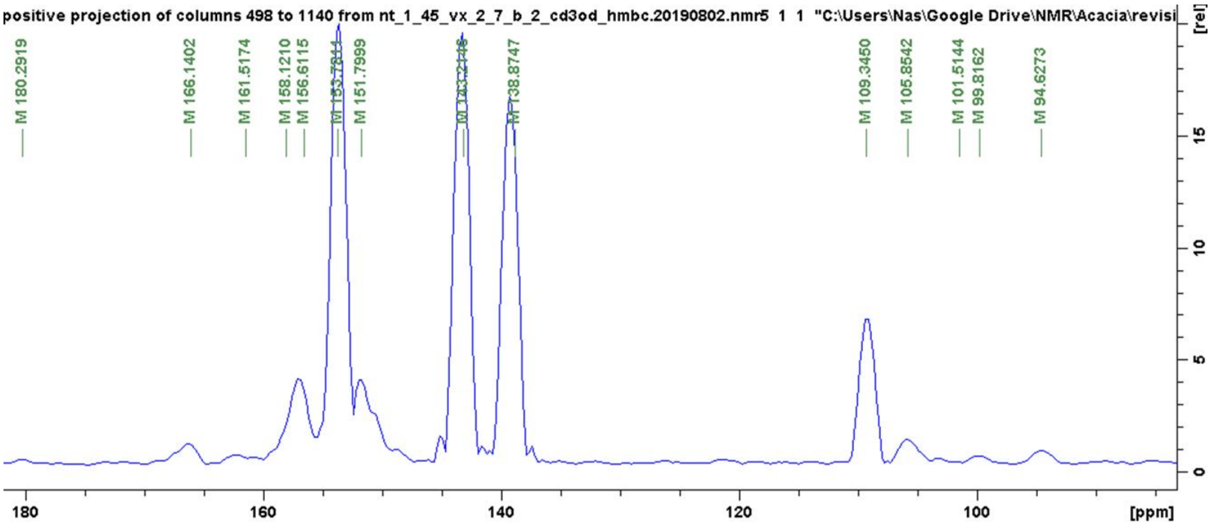

Figure S12. [13]C Spectrum (positive projection from HMBC) of 5,7,2'-trihydroxy-3,4',5'-trimethoxyflavone (3).

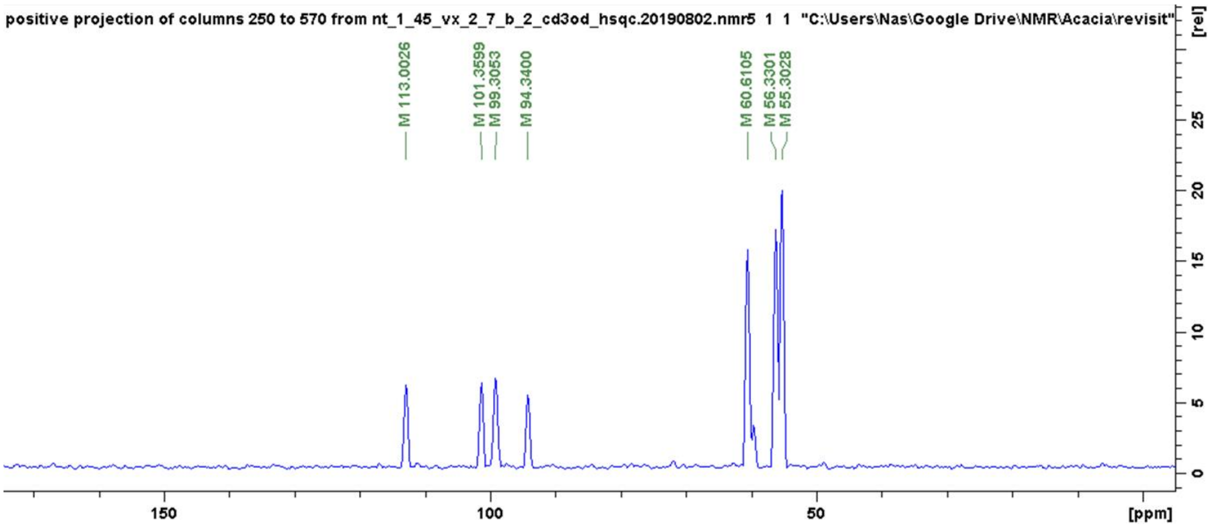

Figure S13. [13]C Spectrum (positive projection from HSQC) of 5,7,2'-trihydroxy-3,4',5'-trimethoxyflavone (**3**).
